# Supplementary material for: CSF1R regulates the dendritic cell pool size in adult mice via embryo-derived tissue-resident macrophages
Source: Nat Commun. 2018 Dec 11;9:5279. doi: 10.1038/s41467-018-07685-x (PMC6290072; doi:10.1038/s41467-018-07685-x)
Supplement: Supplementary file 1 — Supplementary Information [file 41467_2018_7685_MOESM1_ESM.pdf]

Reviewers' comments:

Reviewer #1 (Remarks to the Author):

This study demonstrates that CSF1R controls the DC pool size in FLT3-deficient mice. Remarkably, CSF1R does not act in a cell intrinsic fashion. In fact, it acts by promoting the development of embryo-derived spleen-resident macrophages, which control the differentiation of DC.
The novelty of this paper is two-fold
1. It provides a novel regulatory pathway to control the differentiation of mature DC
2. it shows a novel function of spleen-resident macrophages i.e. supporting and maintaining the pool of DC in FLT3-deficient mice.
The paper is well organized and clearly written. The experiments are convincing and state-of-the-art. Notably, the authors have generated a number of conditional KO mice and reporters to support their conclusions in vivo.
I have only minor suggestions that the authors may address:
1. It is unclear why this novel mechanism unveils only in FLT3-deficient mice. Is this a redundant mechanism which becomes necessary only in the absence of FLT3? The authors should comment on the redundancy of this novel pathway
2. There is little speculation about the mechanisms by which spleen-resident macrophages control the size of the splenic DC pool. The authors should expand the discussion of this important point.

Reviewer #2 (Remarks to the Author):

“CSF1R regulates the dendritic cell pool size in adult mice via embryo-derived tissue-resident macrophages” by Percin et al provides interesting results regarding the regulation of the splenic DC pool via embryo-derived red pulp-MFs. This concept of cell-extrinsic regulation of the DC pool by a non-hematopoietic derived cell type is novel and could be of interest for researchers from different fields. Although we believe the questions addressed in this manuscript are relevant and well investigated, some additional experiments or figures should be provided in the revised manuscript. In addition, several adjustments should be made to the text in order to clarify and present the results obtained in this study in a better way.

Major comments that should be addressed:
1. The effects of CSF1R depletion are only apparent in the absence of Flt3. How biologically relevant is this mechanism and could you explain (or at least speculate on the reason for) this finding in more detail. The authors could use another mouse model in which DCs are lacking (one or both subsets) to see whether this effect is reproducible. The LPS experiment in figure 6 does give some information regarding this topic but does involve clodronate liposomes and inflammation, which both could have additional effects.
2. I have a major concern regarding the littermate controls that were used in this paper. Flt3+/- and Csf1r+/- are used instead of true WT littermate controls. This should at least be addressed or a comment should be added in the text or Materials and Methods to clarify this. This is a bit confusing for the reader. One figure showing that these mice have similar effects would be sufficient, but make sure it is clear for the reader that in the rest of the paper heterozygous KO mice are used as littermate controls. Many studies in the past have shown that heterozygous deletion of a gene can have tremendous effects.
In line with this, same comment holds true for Fig 2 where Fl/+ are used in comparison with Fl/- mice.
3. In Fig 1, it would be useful for the reader if the different macrophage subsets are already introduced here. It’s not clear why they only look here at the F4/80low cells and not yet at the RP-MFs. This should be adjusted or clarified in the text. RP-MFs should already be shown here.
4. Fig 1: Although the data on MDPs are nice, additional data on the CDP population would be interesting. It is mentioned in the text that Pre-cDC1s and Pre-cDC2s are not affected, but this

data is not shown. It should be mentioned as (data not shown) or the data should be presented.
5. Although mentioned in the discussion that adult RP-MFs do not longer require CSF1R, I do not find a figure in this paper showing that (3week old) mice lacking CSF1R do or don't have RP-MFs. As such, the authors do not provide sufficient evidence for the claim that RP-MFs become independent of CSF1R signaling for their generation/maintenance. This figure should be added (on an Flt3-/- background).

Minor comments:
1. Abstract: "combining Flt3 and CSF1R-deficiencies results in specific and complete abrogation of spleen DCs in vivo". Based on Fig 1a, I would not call this effect "specific", you clearly see from the Flow data that other cell types are affected, although this is not commented in the text. This should also be changed in the text related to figure 1. Moreover, in sup Fig1b, you do see an effect on macrophages.
2. Introduction: "Csf1r null mice show normal Dc differentiation and numbers in peripheral organs". Although different mice were used, Macdonald et al JI 2015 show that op/op mice do have reduced splenic DCs, could be important reference to add as this does fit with results later presented in the paper, showing that it could at least be partially explained via CSF1 and not by the other ligand.
3. Figure 1: "CD8+ or CD11b+ DC subsets were affected equally". This data is not shown in the paper and as such, this should be mentioned as (data not shown).
4. In the text related to supplementary Fig 1, there are some mistakes regarding the numbering of the figures as this does not fit with the text. Please adjust this and check also for other figures!
5. There is never a reference to supplementary Fig 1e in the text. This is also the case for supplementary fig 3b-d. Please adjust this. They are mentioned in the legend of the figures but it would be nice to also add them to main text.
6. Clondronate liposomes are known to affect more than just MFs and cause a certain degree of inflammation and cell infiltration. The caveats of this system (although the proper controls are integrated and they look at a time point that most cells have recovered) should be mentioned in the text or discussion. We acknowledge that at this stage, no better techniques are available to specifically deplete RP-MFs.

Point-by-point response to the referees:

Reviewer #1

This referee recognizes the novelties of our study and states that it is 'remarkable' that 'CSF1R does not act in a cell intrinsic fashion' but that, instead, CSF1R-mediated signals 'act by promoting the development of embryo-derived macrophages, which control the differentiation of spleen-resident macrophages, which control the differentiation of DCs.' He further acknowledges that the data presented in our manuscript makes two novel points: '1. novel regulatory pathway to control the differentiation of DCs, 2. novel function of spleen-resident macrophages'. We greatly appreciate these comments. He/she raises two important concerns that we have addressed.

Minor points:

1. It is unclear why this novel mechanism unveils only in FLT3-deficient mice. Is this a redundant mechanism which becomes necessary only in the absence of FLT3? The authors should comment on the redundancy of this novel pathway

This is an important point. We now provide the following text in the discussion section of the revised manuscript.

'In Flt3-deficient mice spleen DC numbers are reduced due to a cell-intrinsic requirement for FLT3 for homeostatic DC division. Csf1r- or Csf1-deficient mice have normal or mildly reduced numbers of spleen DCs, respectively, and only the combined deficiency of Flt3 and Csf1r results in the complete absence of spleen DCs in situ. We show here that cell extrinsic support for DC differentiation becomes evident and important exclusively under stress situations. These stressors can be FLT3-deficiency, which is experimental stress during development (Figure 1) and in adult mice (Figure 5), or the need for rapid regeneration after activation-induced depletion in situ, which provides a physiological challenge in adult mice (Figure 6). Thus, only in the context of the establishment or maintenance of a normally sized DC pool the CSF1R-activated pathway is crucial. The support provided by CSF1R-mediated signals can be seen as a novel kind of redundancy where the effects of the supporting receptor targets a different cell type and thereby works by a cell-extrinsic mechanism. This may serve as a support system to ensure the presence of immune regulatory DCs in situ. Taken together our data link blood cell differentiation from adult HSCs to a cell type of a different ontogeny, providing a novel regulatory principle for innate immune cell differentiation. The interdependency between cells of different ontogenies may be just an example and the differentiation of other cells of the mononuclear phagocyte system and maybe also of adaptive immune cells may depend on similar principles.'

2. There is little speculation about the mechanisms by which spleen-resident macrophages control the size of the splenic DC pool. The authors should expand the discussion of this important point.

We expanded the discussion section with the following statements:

'The precise mechanism of cross talk between RP-Mp and DCs remains unknown. However, using an in vitro differentiation assay, spleen cells enhance the differentiation of DCs in vitro and this support was found independent of direct cell-cell contact, suggesting that a soluble

factor may be the molecular mediator of that support.'

Reviewer 2

This referee thinks that our study is 'interesting' and acknowledges that the 'concept of cell-extrinsic regulation of the DC pool by a non-hematopoietic derived cell type is novel and could be of interest for researchers from different fields'. However, he/she raises several concerns that we have all addressed:

Major points:

1. The effects of CSF1R depletion are only apparent in the absence of Flt3. How biologically relevant is this mechanism and could you explain (or at least speculate on the reason for) this finding in more detail. The authors could use another mouse model in which DCs are lacking (one or both subsets) to see whether this effect is reproducible. The LPS experiment in figure 6 does give some information regarding this topic but does involve clodronate liposomes and inflammation, which both could have additional effects.

We agree with this referee that this is an important point that was also raised by referee #1. We inserted a paragraph on the physiological relevance of our data into the discussion section (please see the response to the first question of referee #1). We think that the support mediated by CSF1R becomes important exclusively in the context of stress situations that require the generation or regeneration of spleen DCs in situ during development or in the adult mouse, respectively.

2. I have a major concern regarding the littermate controls that were used in this paper. Flt3+/- and Csf1r+/- are used instead of true WT littermate controls. This should at least be addressed or a comment should be added in the text or Materials and Methods to clarify this. This is a bit confusing for the reader. One figure showing that these mice have similar effects would be sufficient, but make sure it is clear for the reader that in the rest of the paper heterozygous KO mice are used as littermate controls. Many studies in the past have shown that heterozygous deletion of a gene can have tremendous effects. In line with this, same comment holds true for Fig 2 where Fl/+ are used in comparison with Fl/- mice.

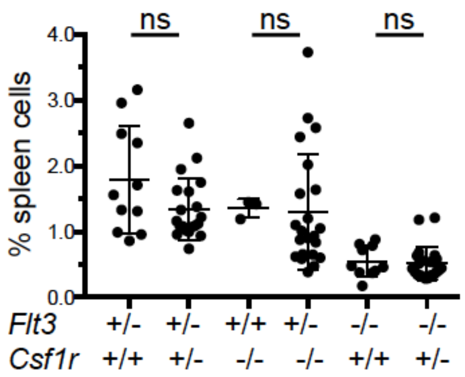

*We agree with this important point. Csf1r$^{+/+}$ and Csf1r$^{+/-}$ mice have the same frequencies of DCs in Flt3$^{+/-}$ (**Figure 1, right**) or Flt3$^{-/-}$ mice (**Figure 1, left**). Flt3$^{+/+}$ and Flt3$^{+/-}$ mice have the same frequencies of DCs in Csf1r$^{-/-}$ mice (**Figure 1, middle**). Based on this data we decided to pool DC data from Flt3$^{+/-}$ and Flt3$^{+/+}$ and Csf1r$^{+/-}$ and Csf1r$^{+/+}$ mice. We inserted this information into the Methods section of the revised manuscript.*

***Figure 1**: DC frequencies in growth factor mutant mice as indicated.*

3.In Fig 1, it would be useful for the reader if the different macrophage subsets are already

introduced here. It's not clear why they only look here at the F4/80low cells and not yet at the RP-MFs. This should be adjusted or clarified in the text. RP-MFs should already be shown here.

We have inserted data on RP-Mps (F4/80^{hi} cells) into Figure 1 of the revised manuscript.

4. Fig 1: Although the data on MDPs are nice, additional data on the CDP population would be interesting. It is mentioned in the text that Pre-cDC1s and Pre-cDC2s are not affected, but this data is not shown. It should be mentioned as (data not shown) or the data should be presented.

We added the data on pre-cDC1s and pre-cDC2s into Supplementary Figure 1 (h-j) and referenced it in the text accordingly.

5. Although mentioned in the discussion that adult RP-MFs do not longer require CSF1R, I do not find a figure in this paper showing that (3week old) mice lacking CSF1R do or don't have RP-MFs. As such, the authors do not provide sufficient evidence for the claim that RP-MFs become independent of CSF1R signaling for their generation/maintenance. This figure should be added (on an Flt3-/- background).

This question is related to question 3 of this referee and the data is integrated in Figure 1 of the revised manuscript. This data is consistent with the lack of effect of CSF1R-deficiency on macrophages subsets described by Cecchini et al. and the reference is cited accordingly. We also included data on the lack of effects of conditional Csf1r depletion in adult mice on RP-Mp into Supplementary Fig. j. In this experimental setting, large peritoneal macrophages were depleted but in the same mice, RP-Mps were not altered (6-8 weeks after induction).

Minor comments:

1. Abstract: 'combining Flt3 and CSF1R-deficiencies results in specific and complete abrogation of spleen DCs in vivo'. Based on Fig 1a, I would not call this effect 'specific', you clearly see from the Flow data that other cell types are affected, although this is not commented in the text. This should also be changed in the text related to figure 1. Moreover, in sup Fig1b, you do see an effect on macrophages.

We relate differences in absolute cell numbers to absolute changes of organ cellularity. This was accomplished by providing the fold-change of organ cellularity and a specific cell type (e.g. Fig. 1b, right for DCs, Fig. 1c, right for macrophages) and show that only DCs are significantly reduced compared to overall loss of spleen cellularity. Macrophage as well as RP-Mp numbers are not reduced when put into relation to overall loss of organ cellularity (Fig. 1c,right, Fig. 1d,right, respectively). Supplemental Fig. 1b shows absolute macrophage numbers that are the basis to calculate the fold-change presented in Fig. 1b,right.

2. Introduction: 'Csf1r null mice show normal Dc differentiation and numbers in peripheral organs'. Although different mice were used, Macdonald et al JI 2015 show that op/op mice do have reduced splenic DCs, could be important reference to add as this does fit with results later presented in the paper, showing that it could at least be partially explained via CSF1 and not by the other ligand.

The reference was included in the text (Material and Methods and Discussion sections).

3. Figure 1: 'CD8+ or CD11b+ DC subsets were affected equally'. This data is not shown in the paper and as such, this should be mentioned as (data not shown).

The data is shown in Supplemental Fig. 1c and referenced in the main text of the revised manuscript.

4. In the text related to supplementary Fig 1, there are some mistakes regarding the numbering of the figures as this does not fit with the text. Please adjust this and check also for other figures!

We apologize for these mistakes – the manuscript was carefully checked throughout and all mistakes were corrected.

5. There is never a reference to supplementary Fig 1e in the text. This is also the case for supplementary fig 3b-d. Please adjust this. They are mentioned in the legend of the figures but it would be nice to also add them to main text.

Both figure parts are now called-out in the main text.

6. Clondronate liposomes are known to affect more than just MFs and cause a certain degree of inflammation and cell infiltration. The caveats of this system (although the proper controls are integrated and they look at a time point that most cells have recovered) should be mentioned in the text or discussion. We acknowledge that at this stage, no better techniques are available to specifically deplete RP-MFs.

The information was included in the main text.

REVIEWERS' COMMENTS:

Reviewer #1 (Remarks to the Author):

I am satisfied with the authors' revision and comments.

Reviewer #2 (Remarks to the Author):

The authors have satisfactorily responded to my concerns. This is a nice story.

REVIEWERS' COMMENTS:
Reviewer #1 (Remarks to the Author):
I am satisfied with the authors' revision and comments.
Reviewer #2 (Remarks to the Author):
The authors have satisfactorily responded to my concerns. This is a nice story.

We are delighted that both referees are satisfied with our responses and we appreciate that they both find merit in the manuscript.
